# Marine Collagen Peptides Promote Cell Proliferation of NIH-3T3 Fibroblasts via NF-κB Signaling Pathway

**DOI:** 10.3390/molecules24224201

**Published:** 2019-11-19

**Authors:** Fei Yang, Shujie Jin, Yunping Tang

**Affiliations:** 1 School of Nursing, Zhejiang Chinese Medical University, Hangzhou 310053, China; yangfei19870826@126.com; 2 Zhejiang Provincial Engineering Technology Research Center of Marine Biomedical Products, School of Food and Pharmacy, Zhejiang Ocean University, Zhoushan 316022, China; m18868006087@163.com

**Keywords:** *Nibea japonica*, marine collagen peptides, proliferation, wound healing, processing by-products

## Abstract

Marine collagen peptides (MCPs) with the ability to promote cell proliferation and migration were obtained from the skin of *Nibea japonica*. The purpose of MCPs isolation was an attempt to convert the by-products of the marine product processing industry to high value-added items. MCPs were observed to contain many polypeptides with molecular weights ≤ 10 kDa and most amino acid residues were hydrophilic. MCPs (0.25–10 mg/mL) also exhibited 2, 2-diphenyl-1-picrylhydrazyl (DPPH), hydroxyl, superoxide anion, and 2′-azino-bis-3-ethylbenzothiazoline-6-sulfonic acid (ABTS) radical scavenging activities. Furthermore, MCPs promoted the proliferation of NIH-3T3 cells. In vitro scratch assays indicated that MCPs significantly enhanced the scratch closure rate and promoted the migration of NIH-3T3 cells. To further determine the signaling mechanism of MCPs, western blotting was used to study the expression levels of nuclear factor kappa-B (NF-κB) p65, IκB kinase α (IKKα), and IκB kinase β (IKKβ) proteins of the NF-κB signaling pathway. Our results indicated protein levels of NF-κB p65, IKKα and IKKβ increased in MCPs-treated NIH-3T3 cells. In addition, MCPs increased the expression of epidermal growth factor (EGF), fibroblast growth factor (FGF), vascular endothelial growth factor (VEGF), and transforming growth factor (TGF-β) in NIH-3T3 cells. Therefore, MCPs, a by-product of *N. japonica,* exhibited potential wound healing abilities in vitro.

## 1. Introduction

Owing to the boom in marine product processing industry, there is a huge production of by-products that are either discarded or simply used as animal feed or fertilizer [1,2]. Hence, there is an urgent requirement to explore methods for using such by-products to yield high value-added items. Bioactive peptides from marine resources possess several physiological functions, including antioxidative [3], anticancer [4], antibacterial [5], angiotensin-converting-enzyme (ACE) inhibitory [6], immunomodulatory [7], hepatoprotective [8], and wound healing activities [9]. Therefore, extraction of bioactive peptides from marine wastes and by-products might offer new avenues for their utilization, consequently preventing environmental pollution and creating enormous economic benefits [6,10].

Collagen extracted from marine by-products is in demand due to the absence of religious restrictions on its use, low immunogenicity, and non-cytotoxicity [11,12]. Marine collagen undergoes enzymatic and chemical hydrolysis to generate marine collagen peptides (MCPs). [13]. Compared to collagen, MCPs possess several advantages, such as ease of absorption for its lower molecular weight and unique physiological functions (including antioxidation) [3,14], high affinity to calcium [15], antihypertensive [16,17], and wound healing activities [18,19].

Currently, the effects of collagen peptides or their combinations with other functional ingredients on wound healing have been the focus of many studies because of their outstanding antioxidant and antimicrobial properties [13,18]. Wound healing is a complex process involving cell-matrix interactions, inflammation, new tissue formation and tissue remodeling [19,20]. Fibroblasts are responsible for regeneration and remodeling of connective tissue in healthy skin [18,20]. So far, there are only few studies dedicated to explore the mechanism of wound healing induced by MCPs in vitro or in vivo. Previous researches have shown a close association between wound healing and nuclear factor kappa enhancer binding protein (NF-κB) signaling pathway [5,21]. NF-ĸB is related to cell proliferation, cell adhesion, inflammation and elimination of reactive oxygen species (ROS) [20]. In addition, the NF-ĸB signaling pathway has been reported to be involved with cutaneous [7,22] and corneal epithelial wound healing [21]. As NF-ĸB signaling and MCPs are both connected to wound healing, we hypothesized that this pathway might play an important role in wound healing induced by MCPs.

Previously, marine collagen was successfully obtained from the skin of *Nibea japonica,* and the physicochemical properties and biocompatibility were determined [11,23]. In an attempt to identify functional MCPs, in the present study, MCPs were extracted from *N. japonica* skins for their functional assessment. We also analyzed the molecular weight, amino acid content and antioxidant activities of the extracted MCPs. Our study showed that MCPs can promote cell proliferation and migration of NIH-3T3 fibroblasts via the NF-ĸB signaling pathway.

## 2. Results and Discussions

### 2.1. Determination of Molecular Weight Distribution of MCPs

**Figure 1:**
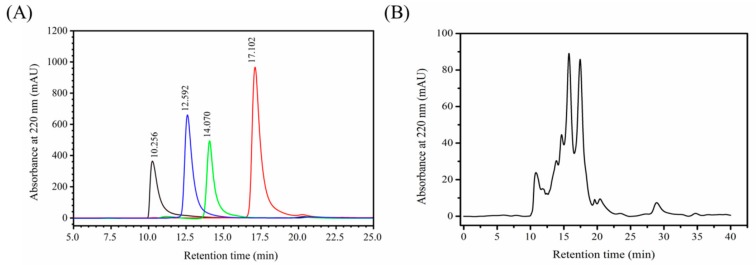
The HPLC spectra of the standard molecular weight samples (**A**) and marine collagen peptides (MCPs) from skin of *Nibea japonica* (**B**).

The HPLC spectrum of the standard molecular weight samples is shown in Figure 1A and the regression equation obtained is as follows:

lgMw = −0.2753Rt + 7.3148(1)

The coefficient of regression (R^2^) was 0.9652 indicating good linear relationship and the molecular weight distribution of MCPs could be determined based on the above equation. The HPLC spectrum of MCPs from the *N. japonica* skins is shown in Figure 1B. Components less than 1, 3, 5 and 10 kDa accounted for 55.25%, 79.29%, 85.71% and 90.31% of the spectrum respectively indicating these MCPs primarily contained a large number of low molecular weight polypeptides. Furthermore, *N. japonica* MCPs had better water solubility than the marine collagen [11] essentially because their low molecular weight structures possess many water-exposed polar amino acid residues, leading to the formation of more hydrogen bonds [24,25].

### 2.2. Amino Acid Content of MCPs

**Figure 2:**
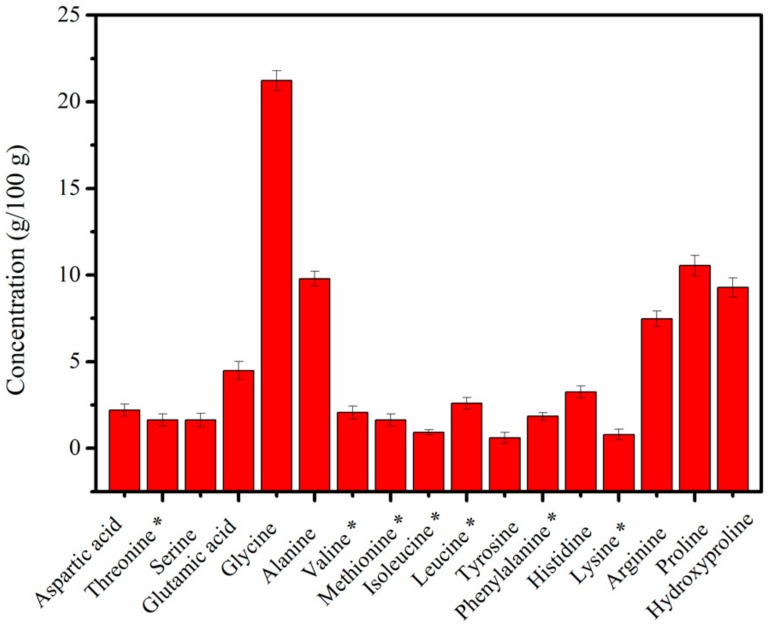
Amino acids content of MCPs extracted from skin of *Nibea japonica*. Note: * essential amino acid. All assays were performed in triplicate.

The amino acid content of MCPs from *N. japonica* skins is shown in Figure 2. The studied MCPs comprised seven essential amino acids (11.49%) and ten non-essential amino acids (70.48%). Glycine was the principal amino acid in MCPs, accounting for approximately 21.22% of the total amino acid composition, followed by proline (10.55%), alanine (9.79%), hydroxyproline (9.28%), arginine (7.47%) and glutamic acid (4.48%). Furthermore, no cysteine was detected in the concerned MCPs. In our previous studies, we confirmed that the collagen from *N. japonica* skins is a type I collagen [11,23]. Cysteine being exclusively present in type III collagen [12] our results confirm our previous observation. MCPs usually contain a high concentration of Gly-Xaa-Yaa triplets, where Xaa is usually proline and Yaa is most likely hydroxyproline [11,12]. The high content of glycine, proline and hydroxyproline in MCPs was consistent with the high frequency of occurrence of the Gly-Pro-Hyp sequence in the collagen. Furthermore, the glycine (21.22%), proline (10.55%), hydroxyproline (9.28%) and arginine (7.47%) contents in MCPs from *N. japonica* skins was similar to that in MCPs from tilapia skin (where the percentage of glycine, proline, hydroxyproline, and arginine were 20.92%, 11.32%, 10.28% and 7.96%, respectively) [13]. The majority of the amino acid residues were hydrophilic, such as hydroxyproline, arginine, glutamic acid, and aspartic acid. This was consistent with the good water solubility of MCPs from *N. japonica* skins.

### 2.3. Antioxidant Activity of MCPs

NADPH oxidase stimulates inflammatory cells to produce large amounts of ROS during the inflammatory phase of wound healing [26,27]. Normally, ROS are scavenged by antioxidants, and there is a balance between ROS production and neutralization [26]. On the contrary, this balance is disturbed in a wound, where excessive ROS are produced. Excessive ROS induction is associated with activation of pro-apoptotic proteins resulting in cell death and necrosis and can be harmful for wound healing. MCPs are widely used in the skin care industry to promote wound healing due to their antioxidant properties and other beneficial properties [28]. Therefore, the antioxidant activities of MCPs (0.25–10 mg/mL) from the skin of *N. japonica* were evaluated using four different radical scavenging assays.

**Figure 3:**
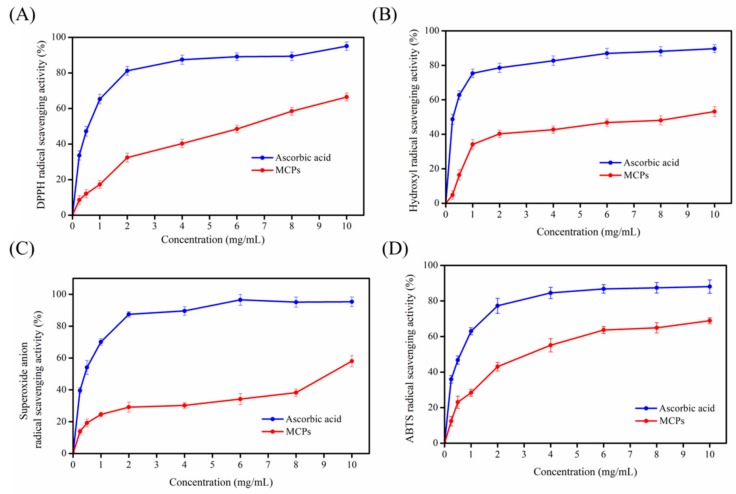
2, 2-diphenyl-1-picrylhydrazyl (DPPH) (**A**), Hydroxyl (**B**), superoxide anion (**C**) and ABTS (**D**) radical scavenging activities of MCP s from *Nibea japonica* skins. All assays were performed in triplicate.

As illustrated in Figure 3, MCPs (0.25–10 mg/mL) obtained from *N. japonica* skins could scavenge DPPH, hydroxyl, superoxide anion and ABTS radicals. The concentration of MCPs was related to the scavenging activities of these four free radicals. The scavenging activities of these four free radicals also increased in proportion to MCPs concentration. However, as shown in Figure 3, the antioxidant activities of MCPs were relatively lower than that of ascorbic acid (approximately 0–60% at concentrations between 0.25 and 10 mg/mL), and should be improved for use in wound healing. Recently, several functional ingredients, such as chitosan, chemically modified chitosan or nicotinamide were used to improve the antioxidant activity of peptides to promote wound healing. For example, the *N*-succinyl chitosan-collagen peptide copolymer manufactured with transglutaminase possessed better antioxidant activity and could be used as a wound healing biomaterial [28]. Nicotinyl-isoleucine-valine-histidine (NA-IVH), manufactured by combining nicotinamide and jellyfish peptides (IVH), showed significant enhancement of radical scavenging function and can promote wound healing under hyperglycemic condition [26]. Therefore, MCPs have to be modified to improve their wound healing properties for subsequent application in wound healing.

### 2.4. Cell Proliferation of NIH-3T3

**Figure 4:**
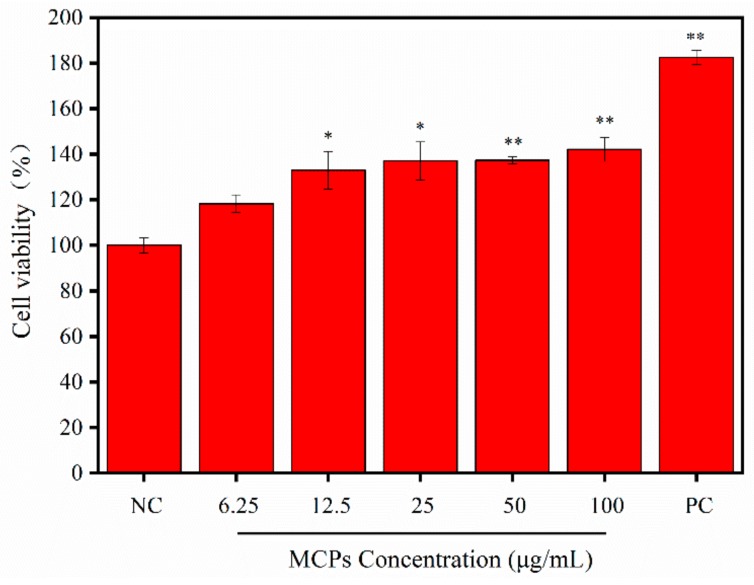
Relative cell viability as affected by 72 h treatment of different concentrations of MCPs from *Nibea japonica* skin. Negative control (NC): adding 0.4% serum DMEM to cells; Experimental group: MCP was dissolved by 0.4% serum DMEM in concentrations of 6.25, 12.5, 25, 50, and 100 μg/mL and then added to the prepared cells; Positive control (PC): adding 10% serum DMEM to cells. * *p* < 0.05 and ** *p* < 0.001 vs. NC. The data were expressed as the mean ± standard deviation (*x* ± *s*, *n* = 6).

Various types of cells are known to undergo migration and proliferation during wound healing. Fibroblasts are the key components of normal wound healing and play an important role from late inflammation to complete epithelialization [29]. The present study demonstrated that MCPs have the potential to promote the growth of NIH-3T3 cells. As shown in Figure 4, the viability rate of NIH-3T3 cells treated with varied concentrations of MCPs increased significantly post 72 h of incubation. The viability of cells treated with 25 μg/mL MCPs was 37% more than that of the negative control (NC) group, but was lower than that of the positive control (PC) group. Our observation is in agreement with the results obtained using MCPs from tilapia, which promoted L929 fibroblast proliferation [18]. Thus, MCPs showed significant proliferation in vitro and have potential to be used for wound healing or cosmetic application.

### 2.5. Effect of MCPs on the Scratch Wound Closure In Vitro

**Figure 5:**
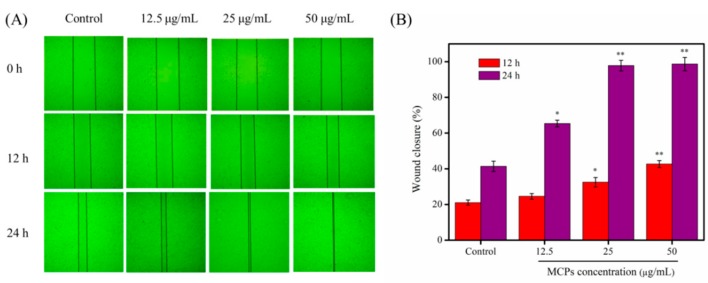
Effect of MCPs from *Nibea japonica* skins on the scratch closure in vitro. (**A**) Representative optical images showed the cells migrated toward wound gap after 12 h and 24 h incubation; (**B**) Wound closure rate (%) that affected by MCPs for 12 h and 24 h. The data was obtained by using Image J 1.38 software and were expressed as the mean ± standard deviation (*x* ± *s*, *n* = 6). * *p* < 0.05 and ** *p* < 0.001 vs. control.

Fibroblast migration can accelerate the process of wound re-epithelialization and promote wound closure during healing [30]. Previously, in vitro scratch test has often been used to simulate wound healing [18,20]. Therefore, we used the above assay on NIH-3T3 cells to evaluate the effect of MCPs from *N. japonica* skins on the wound healing process. As shown in Figure 5, the migration of cells to the scratched area was evident after 12 h and 24 h. In addition, in the presence of MCPs, the wound area was significantly reduced in a dose-dependent manner compared to the control group without MCPs. Significant scratch closure mediated by MCPs was observed after 24 h. In particular, the effect of 50 μg/mL MCPs on in vitro wound healing was highly statistically significant (Figure 5B) and the scratch was almost completely sealed (Figure 5A). Our results indicated that MCPs was capable of inducing NIH-3T3 cell migration and potentially promote wound healing. This may be because abundant amino acids residues in MCPs provide a suitable environment for NIH-3T3 cells to proliferate and migrate (although the mechanism is not clearly delineated).

### 2.6. MCPs Activated the NF-κB Signaling Pathway in NIH-3T3 Fibroblasts

**Figure 6:**
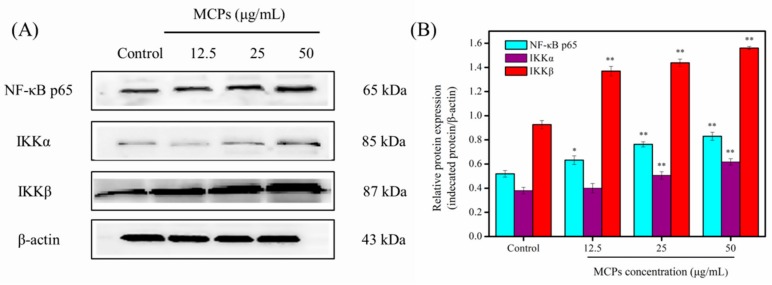
MCPs activated the NF-κB signaling pathway and increased expression of its target pathways in NIH-3T3 fibroblast cells. (**A**) Western blot analysis of the NF-κB p65, IKKα, and IKKβ in the NIH-3T3 cells treated with different concentrations MCPs overnight. (**B**) The expression levels of NF-κB p65, IKKα, and IKKβ analyzed by western blotting. The data was obtained by using Image J 1.38 software and were expressed as the mean ± standard deviation (*x* ± *s*, *n* = 6). * *p* < 0.05 and ** *p* < 0.001 vs. control.

NF-κB is a transcription factor that regulates the expression of multiple genes involved in a variety of cellular functions including cell migration, proliferation, adhesion and survival [20,31]. Therefore, for further confirmation of role of MCPs towards activation of the signaling pathway through NF-κB the protein expression levels of some related proteins were evaluated using western blotting. As shown in Figure 6, NF-κB p65, IκB kinase α (IKKα), and IκB kinase β (IKKβ) levels increased significantly after treatment of different concentrations of MCPs in a dose-dependent manner. These results indicated that MCPs can promote NIH-3T3 cell migration and proliferation via the NF-κB signaling pathway.

### 2.7. Western Blot Analysis of Growth Factors

**Figure 7:**
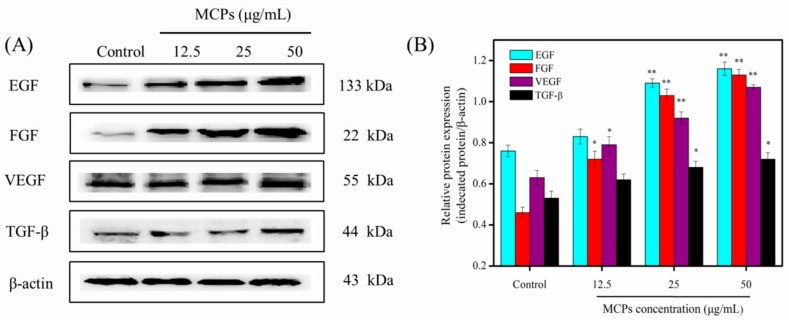
Effect of MCPs on the protein expression levels of epidermal growth factor (EGF), fibroblast growth factor (FGF), vascular endothelial growth factor (VEGF), and transforming growth factor (TGF-β) in NIH-3T3 fibroblast cells. (**A**) Western blot analysis of the EGF, FGF, VEGF, and TGF in the NIH-3T3 cells treated with different concentrations MCPs overnight (**B**) Protein expression levels of EGF, FGF, VEGF, and TGF analyzed by western blotting. The data was obtained by using Image J 1.38 software and were expressed as the mean ± standard deviation (*x* ± *s*, *n* = 6). * *p* < 0.05 and ** *p* < 0.001 vs. control.

Wound healing is a complex process regulated by different signaling pathways, various cytokines and certain growth factors [19,32]. Epidermal growth factor (EGF) can enhance the migration and proliferation of fibroblasts. In addition, EGF promotes angiogenesis and epithelization and triggers growth factor secretion by fibroblasts, which ultimately leads to accelerated wound healing [33]. Fibroblast growth factor (FGF) can enhance angiogenesis, cell migration and proliferation to promote wound healing [34]. Vascular endothelial growth factor (VEGF) is the main growth factor that triggers angiogenesis and stimulates wound healing [20,32]. Transforming growth factor (TGF-β) can also induce various processes such as secretion of extracellular matrix proteins, proliferation, migration, and angiogenesis [35]. Therefore, we further assessed the effects of MCPs from *N. japonica* skin on the expression of EGF, FGF, VEGF, and TGF-β in this study. As shown in Figure 7, the protein levels of EGF, FGF, VEGF, and TGF-β increased significantly after treatment with various concentrations of MCPs. These results support the notion that MCPs can be applied for promoting wound healing. In addition, a continuous over-expression of such growth factors, without a turning-back point towards their initial levels after wound healing, may be linked to other non-beneficial proliferation-related manifestations such as cancerous neoangiogenesis because of an unresolved inflammatory process [36]. So, further in vivo experiments should be applied to the wound surface of the skin to demonstrate the effect of MCPs, and the role of NF-κB signaling pathway or growth factors in promoting wound healing.

## 3. Materials and Methods

### 3.1. Materials

*N. japonica* skins available in our laboratory [11], and the NIH-3T3 fibroblasts were purchased from the Cell Bank of Chinese Academy of Sciences (Shanghai, China). Antibodies raised against NF-κB p65 (cat. No. AF0246), IKKα (cat. No. AF0198), IKKβ (cat. No. AI137), VEGF (cat. no. AF1309), and TGF-β (cat. No. AF0198) were purchased from Beyotime Biotechnology (Shanghai, China). β-actin (cat. no. K200058M) was purchased from Solarbio (Beijing, China). Detection antibodies for EGF (cat. no. 184265) and FGF (cat. no. ab171941) were procured from Abcam (Cambridge, England). MTT cell proliferation and cytotoxicity assay kit (AR1156) was purchased from Boster Biological Technology co. Itd (Wuhan, China). All other reagents were of analytical grade.

### 3.2. Preparation of MCPs from N. japonica skin

The non-collagenous proteins and fat were removed from the *N. japonica* skins following protocol described by Tang et al. [11]. Following it, the fish skins were heated at 100 °C for 10 min, and hydrolyzed in presence of neutral protease (1500 U/g). We adjusted the initial pH of the solution to 7.0 and the enzymatic hydrolysis was performed at 45 °C for 3 h. The above step was followed by enzyme deactivation at 100 °C for 10 min. After centrifugation, the supernatant of MCPs was collected and lyophilized for further study.

### 3.3. Determination of the Molecular Weight Distribution of MCPs

The molecular weight distribution of MCPs was analyzed using high pressure liquid chromatography (HPLC) (Agilent 1200, CA, USA). We used a TSK gel G2000 SWXL analytical column (4.6 × 250 mm, 5 µm) at UV 220 nm at 25 °C, and a mobile phase of acetonitrile/water/trifluoroacetic acid (45:55:0.1) at the flow rate of 0.5 mL/min. The standard samples comprised of peroxidase (40,000 Da), aprotinin (6500 Da), Arg-Val-Ala-Pro-Glu-Glu-His-Pro-Val-Glu-Gly-Arg-Tyr-Leu-Val (1750 Da) [7], and Tyr-Val-Pro-Gly-Pro (530 Da) [4] which were loaded into the column by turn. The standard curve of retention time and absorbance was plotted. The MCPs solution was then filtered using 0.22 μm micropore film and injected under the same conditions. Finally, the molecular weight distribution of MCPs was calculated according to the standard curve equation.

### 3.4. Amino Acid Content

The amino acid content was determined according to the Chinese national standard (GB5009124-2016). MCPs were first dissolved in 6 M HCl solution and hydrolyzed at 110 °C for 24 h. The hydrolysate was further diluted with citric acid buffer and analyzed using an amino acid analyzer L-8900 (Hitachi, Tokyo, Japan). The hydroxyproline content was analyzed as per procedure described by Tang et al. [11].

### 3.5. Antioxidant Activity of MCPs

The hydroxyl, 2, 2-diphenyl-1-picrylhydrazyl (DPPH), 2, 2′-azino-bis-3-ethylbenzothiazoline-6-sulfonic acid (ABTS), and superoxide anion radical scavenging activity of MCPs was analyzed according to Zhao et al. [37].

### 3.6. Proliferation of NIH-3T3 Fibroblasts in Presence of MCPs

The MTT assay was employed to assess proliferation of MCPs [11]. Briefly, NIH-3T3 cells were seeded in a 96-well plate at a density of 2 × 10^5^ cells/mL and cultured in complete medium (Dulbecco’s modified Eagle’s medium (DMEM) supplemented with 10% fetal bovine serum (FBS), 100 U/mL penicillin and 100 μg/mL streptomycin) at 37 °C in a 5% CO_2_ incubator. Upon 80–90% cellular confluence the culture medium was substituted with fresh maintenance medium (DMEM contains 0.4% FBS), and the cells were further grown for duration of 24 h at 37 °C. Further the cells were exposed to different concentrations of MCPs (0, 6.25, 12.5, 25, 50 and 100 µg/mL) in the maintenance medium, and cultured for 24, 48 and 72 h respectively. Cells grown in the same volume of complete medium served as the positive control group. The optical density (OD) at 490 nm was determined using a microplate reader (SpectraMa, Molecular Devices Co., San Jose, CA, USA), and relative cell viability (%) was calculated using the following formula:

Relative cell viability (%) = [1 − (OD treated/OD untreated)] × 100%(2)

### 3.7. In Vitro Scratch Wound Assay

NTH-3T3 cells were seeded in a 6-well plate at a density of 2 × 10^5^ cells/mL and incubated in complete medium until cell confluence reached about 80–90%. The cells were further grown for next 24 h at 37 °C in 5% CO_2_ incubator. A uniform scratch wound was created using a 200 μL sterile pipette tip, and the wound debris was removed through phosphate buffer saline (PBS) wash. The scratched cells were then treated with different concentrations of MCPs (0, 12.5, 25 and 50 µg/mL) and cultured for 12 or 24 h. Scratch closure was evaluated using an inverted microscope (Olympus, Tokyo, Japan) and the scratch area was analyzed using the Image J 1.38 software (NIH, Bethesda, MD, USA). We enumerated the scratch closure rate (%) based on the following formula:

Scratch closure rate (%) = (A_0_ − A_t_)/A_0_ × 100% (3)

where A_0_ represents the scratch area at 0 h and A_t_ represents the same at the designated time point.

### 3.8. Western Blot Analysis

Western blotting of target proteins helped to confirm the proliferation of MCPs on NIH-3T3 cells., We used the technique according to Jiang et al. [38] with certain modifications. We used a seeding density of 2 × 10^5^ cells/mL for culturing NTH-3T3 cells and treated with varied concentrations of MCPs (0, 12.5, 25 and 50 µg/mL) for 24 h. Subsequently, the cells were collected and lysed in radioimmunoprecipitation assay (RIPA) lysis solution. Protein concentration of cellular lysates was obtained using the bicinchoninic acid (BCA) protein assay. Further, an equivalent amount of denatured protein sample (30 μg) was resolved using 12% sodium dodecyl sulfate (SDS)-polacrylamide gel. After electrophoresis, the gel was transferred onto a polyvinylidene difluoride (PVDF) membrane. Non-specific binding was prevented through incubation with 5% skimmed milk for 1 h followed by overnight incubation with diluted (1:1000) primary antibodies (NF-κB p65, IKKα, IKKβ, VEGF, EGF, FGF, and TGF-β) at 4 °C. We finally incubated the membranes in diluted (1:1000) secondary antibodies for 1 h at room temperature. The target protein bands were visualized using enhanced chemiluminescence and the density was enumerated using the Image J 1.38 software (NIH, Bethesda, MD, USA). We used β-Actin as an internal control.

### 3.9. Statistical Analysis

We represented all experimental data as the mean ± standard deviation (*x* ± *s*, *n* = 6) and analyzed using the SPSS software version 24.0 (SPSS Inc., Chicago, IL, USA). Statistical significance of the data was determined using one-way analysis of variance (ANOVA).

## 4. Conclusions

In the present study, MCPs prepared from the skin of *N. japonica* exhibited potential cell proliferation and migration activities. Our results indicated that MCPs are rich in polypeptides with molecular weights ≤ 10 kDa. MCPs could scavenge DPPH, hydroxyl, superoxide anion, and ABTS radical as well as promoted the proliferation and migration of NIH-3T3 cells. In vitro scratch assays also reflected that MCPs significantly affect the scratch closure rate. MCPs further increased the protein levels of NF-κB p65, IKKα, and IKKβ, which are prominent members of the NF-κB signaling pathway, as well as those of certain growth factors such as EGF, FGF, VEGF, and TGF-β in NIH-3T3 cells (as revealed through western blotting). In conclusion, our results indicated that MCPs from the skin of *N. japonica* possess potential to promote wound healing. The findings may provide guidance for high value-added utilization by-products of marine processing industry. In the future, in vivo experiments are needed to apply to the wound surface of the skin to demonstrate role of MCPs in promoting wound healing.
